# PIWIL2 suppresses Siah2-mediated degradation of HDAC3 and facilitates CK2α-mediated HDAC3 phosphorylation

**DOI:** 10.1038/s41419-018-0462-8

**Published:** 2018-03-19

**Authors:** Yingying Zhang, Xulei Zheng, Hao Tan, Yilu Lu, Dachang Tao, Yunqiang Liu, Yongxin Ma

**Affiliations:** Department of Medical Genetics, State Key Laboratory of Biotherapy, West China Hospital, Sichuan University and Collaborative Innovation Center, Chengdu, 610041 China

## Abstract

HDAC3 is involved in deacetylation of histone and non-histone proteins, having a key role in the regulation of gene transcription and also in the process of tumorgenesis. However, how HDAC3 is regulated in cancer remains largely unclear. Here, we showed that PIWIL2 can interact with HDAC3, leading to stabilization of HDAC3 from ubiquitin-mediated degradation by competitive association with E3 ubiquitin ligase Siah2. Furthermore, we found that expression of PIWIL2 enhanced HDAC3 activity via CK2α. PIWIL2 facilitated the interaction between HDAC3 and CK2α, thus exhibiting a promotion on the HDAC3 phosphorylation by CK2α. Further work showed that PIWIL2 could promote cell proliferation and suppress cell apoptosis via regulating HDAC3. Our present study firstly revealed that PIWIL2 can play a role in HDAC3-mediated epigenetic regulation on cancer cell proliferation and apoptosis. These findings provide a novel insight into the roles of PIWIL2 in tumorigenesis.

## Introduction

Histone deacetylases (HDACs), which can remove acetyl from lysine residues and induce transcriptional repression, play an important role in gene regulation and chromatin structure, showing various effects on metabolism and cancer^1–4^. HDACs can be divided into four classes, class I, II, III (sirtuins), and IV, based on their catalytic mechanism and sequence homology^5,6^. HDACs belonging to Class I, II, and IV require zinc mental for enzymatic activities, whereas class III HDACs (sirtuins) need nicotine adenine dinucleotide as a cofactor. Consideration of their important roles in cancer, inhibitors of HDACs, such as butyrate, trichostatin A (TSA) and vorinostat can be used as anti-cancer agents^7–9^. HDACs are involved in the deacetylation not only of histone proteins, but also of non-histone substrates, such as p53, YY1 GATA-2, and NF-kB^10^. Generally, hypoacetylation of histone proteins are associated with repression of gene expression, whereas hyperacetylation are associated with increased transcriptional activity^11–13^.

HDAC3, a member of the class I HDAC family, is overexpressed in many cancer cells^14^. HDAC3 is found in the nucleus, cytoplasm, and plasma membrane, while HDAC1 and HDAC2 predominantly in nucleus^5,15,16^. Previous studies showed that HDAC3 inhibited P53, P27, Bax gene transcriptions via H3K9 deactylation, and reduced basal and butyrate-induced p21 expression. HDAC3 inhibition can induce expression of alkaline phosphatase (AP, a marker of colon cell maturation), trigger degradation of c-Myc protein and reduce the stability of DNMT1 protein^14,17,18^, indicating that HDAC3 plays an important role in cancer cell proliferation and apoptosis. Currently, it is demonstrated that the activity of HDAC3 was modulated by two distinct mechanisms. One is interaction with multisubunit protein complex that contain NCoR and SMRT; the other is through its phosphorylation or dephosphorylation^19–21^. However, how HDAC3 is regulated in cancer remains largely unknown.

PIWIL2 (aka hili in humans or mili in mouse) is a member of PIWI family, which is defined by highly conserved PAZ and PIWI domains^22^. PIWIL2 could not be found in normal tissues except germ cells of testis in normal adults, but it is widely expressed in various types of tumors, including gastrointestinal, breast, ovarian, and endometrial cancer^23–26^. Our previous study showed that PIWIL2 plays roles in tumorigenesis and tumor development through several underlying mechanisms. PIWIL2 promoted cancer cell proliferation via increasing c-Myc expression by facilitating NME2 binding to the G4-motif, facilitated cancer cell migration via TBCB and resisted Fas-induced cancer cell apoptosis by inhibiting keratin 8 degradation^27–29^. In our previous study, we found that PIWIL2 could bind to specific location of gene by associating with some specific transcription factors to regulate gene expression^27^. So we are curious about whether PIWIL2 exerted a role in cancer through an association with epigenetic factors.

Here we present that PIWIL2 interacts with HDAC3 and promotes the stability of HDAC3. Besides, PIWIL2 increases the phosphorylation of HDAC3 by promoting CK2α to phosphorylate HDAC3. Our current study revealed a novel role that PIWIL2 plays a role in epigenetic regulation in tumorigenesis.

## Results

### PIWIL2 binds with HDAC3 specifically in class I HDACs

**Fig. 1:**
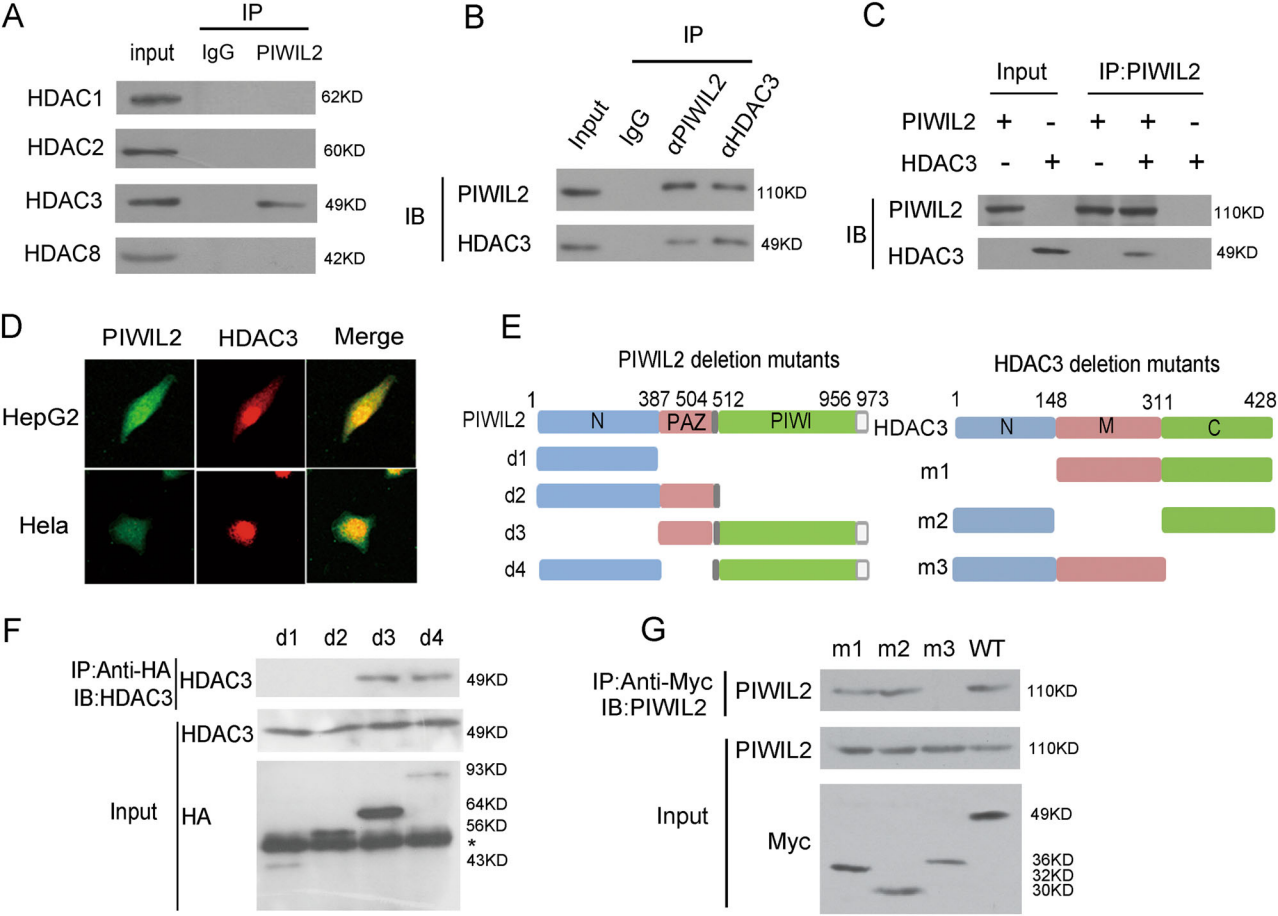
PIWIL2 interacts with HDAC3. **a** PIWIL2 can only interact with HDAC3 among class I HDACs. **b** Endogenous interaction between PIWIL2 and HDAC3. **c** PIWIL2 binds to HDAC3 in a TNT^®^ Quick Coupled Transcription/Translation System. **d** Co-localization of PIWIL2 and HDAC3 using immunofluorescence assays. **e** Schematics of PIWIL2 deletion mutants and HDAC3 deletion mutants. **f** PIWI domain is necessary for PIWIL2 binding with HDAC3. The “ *” indicates a non-specific bind. **g** C-terminal region of HDAC3 is necessary for HDAC3 binding with PIWIL2

To analyze the putative interaction of PIWIL2 with different members of the class I family of HDACs, cell lysates were subjected to immunoprecipitation (IP) with anti-PIWIL2 antibody and analyzed by Western blotting (WB). Results showed that only HDAC3 could interact with PIWIL2 among all these four HDACs (Fig. 1a). To further validate this interaction, we also carried out the IP with anti-PIWIL2 or anti-HDAC3 antibodies respectively. The endogenous PIWIL2 and HDAC3 can bind with each other (Fig. 1b). Furthermore, the physical interaction between PIWIL2 and HDAC3 was analyzed by a TNT Quick Coupled Transcription/Translation Systems in vitro (Fig. 1c).

We next detected the putative cellular co-localization of PIWIL2 with HDAC3 by immunofluorescence. Unlike other class I family of HDACs whose localization is only in nucleus, HDAC3 localized both in nucleus and cytoplasm^5^. As shown in Fig. 1d, PIWIL2 co-localized with HDAC3 in both nucleus and cytoplasm, mainly in nucleus.

To further identify the domain(s) of PIWIL2 or HDAC3 responsible for the PIWIL2-HDAC3 interaction, a series of deletion mutants of PIWIL2 or HDAC3 were used for detection (Fig. 1e). Cells were transfected with deletion mutants of HA-PIWIL2 or Myc-HDAC3 followed by Co-IP experiments. As shown in Fig.1f, PIWIL2 mutants without PIWI domain but not N-terminal or PAZ domain failed to interact with HDAC3. N-terminal deletion mutant or deletion mutant of the middle segment of HDAC3 can bind with PIWIL2, whereas C-terminal deletion mutant can’t interact with PIWIL2 (Fig. 1g). The results indicate that C terminus is necessary for HDAC3 to interact with PIWIL2.

### PIWIL2 stabilizes HDAC3 protein

**Fig. 2:**
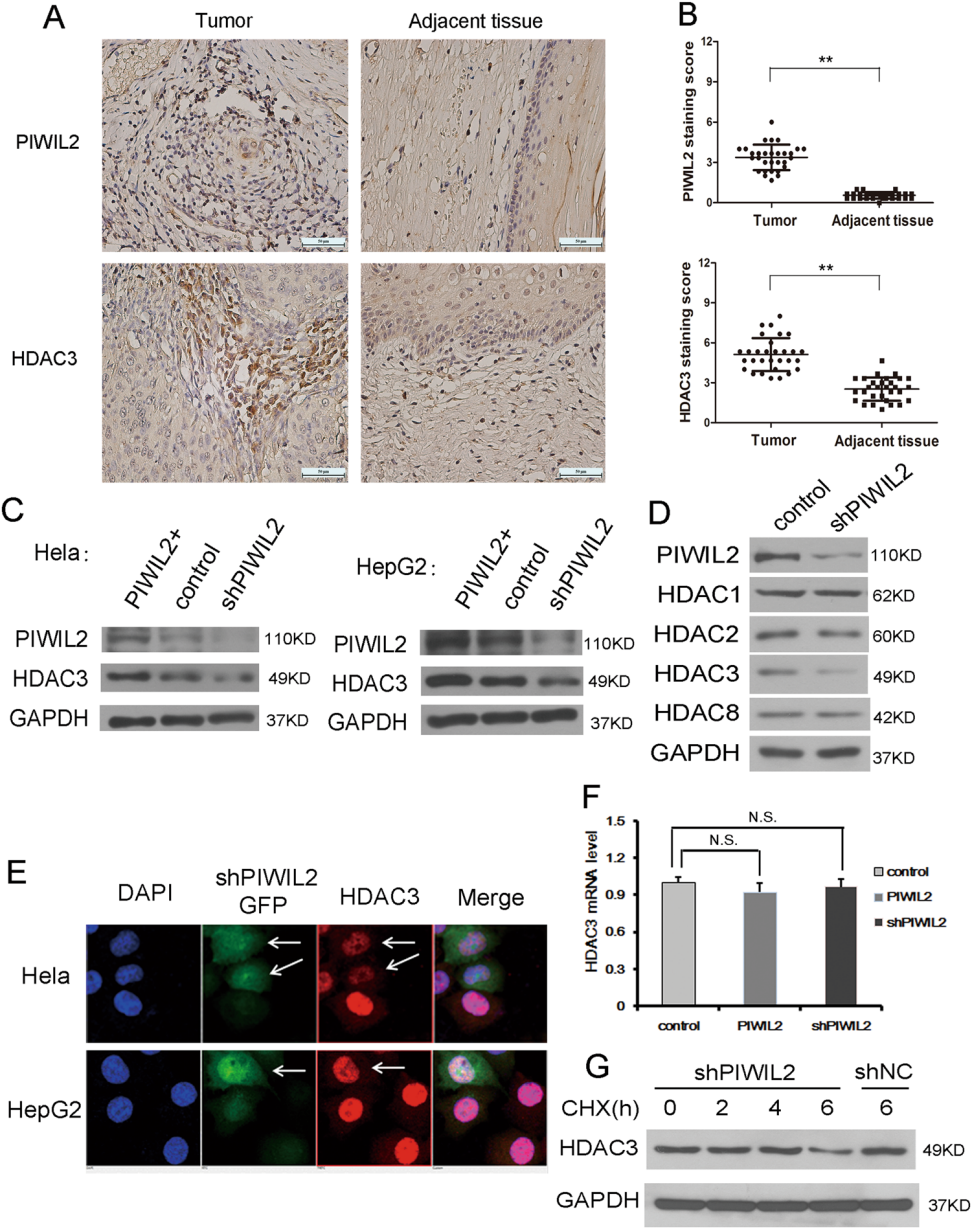
PIWIL2 stabilizes HDAC3 protein. **a**, **b** Expression of PIWIL2 and HDAC3 in tumor and adjacent normal tissues from 30 pairs of cervical cancer patients were examined. The magnification is ×400. *P < 0.05 and **P < 0.01. **c** PIWIL2 up-regulates HDAC3 expression at protein level in Hela cells and HepG2 cells. **d** Knockdown of PIWIL2 specifically decreases expression of only HDAC3 among class I HDACs. **e** Immunofluorescent staining of HDAC3 in PIWIL2 shRNA transfected Hela and HepG2 cells. **f** PIWIL2 has no effect on HDAC3 mRNA expression (N.S., not significant, *P* > 0.05). **g** The degradation of HDAC3 is faster in cells transfected with PIWIL2 shRNA than in cells transfected with control plasmid. Cells transfected with shPIWIL2 or shNC were treated with cycloheximide (CHX) at 50 μM for indicated time

We analyzed the expression of PIWIL2 and HDAC3 by tissue microarray containing 30 pairs of cervical tumor tissues and adjacent normal tissues, and found that both PIWIL2 and HDAC3 were more highly expressed in tumor tissues than those in adjacent tissues (Fig. 2a, b). More interestingly, the decrease of PIWIL2 induced a reduction of HDAC3 when we knocked down PIWIL2 with specific shRNA (Fig. 2c). Consistently, when PIWIL2 levels were overexpressed in Hela or HepG2 cells, endogenous HDAC3 level was higher than that in control group. To determine whether this regulation of PIWIL2 on HDAC3 was specific, we studied the effects of decreased level of PIWIL2 on class I family of HDACs by shRNA. Results indicated that PIWIL2 influenced the level of HDAC3 but not HDAC1, HDAC2 or HDAC8 (Fig. 2d).

Immunofluorescence using antibody against HDAC3 was performed with a confocal microscope to determine the protein level changes when PIWIL2 was knocked down. All images were taken under the same exposure times for comparison. Lower fluorescence intensity of HDAC3 was observed in Hela or HepG2 cells transfected with shPIWIL2 compared with control group (Fig. 2e). Taking into account that PIWIL2 may regulate HDAC3 in its transcriptional level, we studied the mRNA level of HDAC3 after increasing or decreasing PIWIL2 level. But no significant difference of mRNA level was observed between these groups (Fig. 2f). Thus, PIWIL2 regulates the level of HDAC3 not in a transcription-dependent manner. Cells were then treated with protein synthesis inhibitor CHX, and the WB result showed that PIWIL2 knockdown accelerated the degradation of HDAC3 protein (Fig. 2g).

### PIWIL2 inhibits the Siah2-mediated degradation of HDAC3

**Fig. 3:**
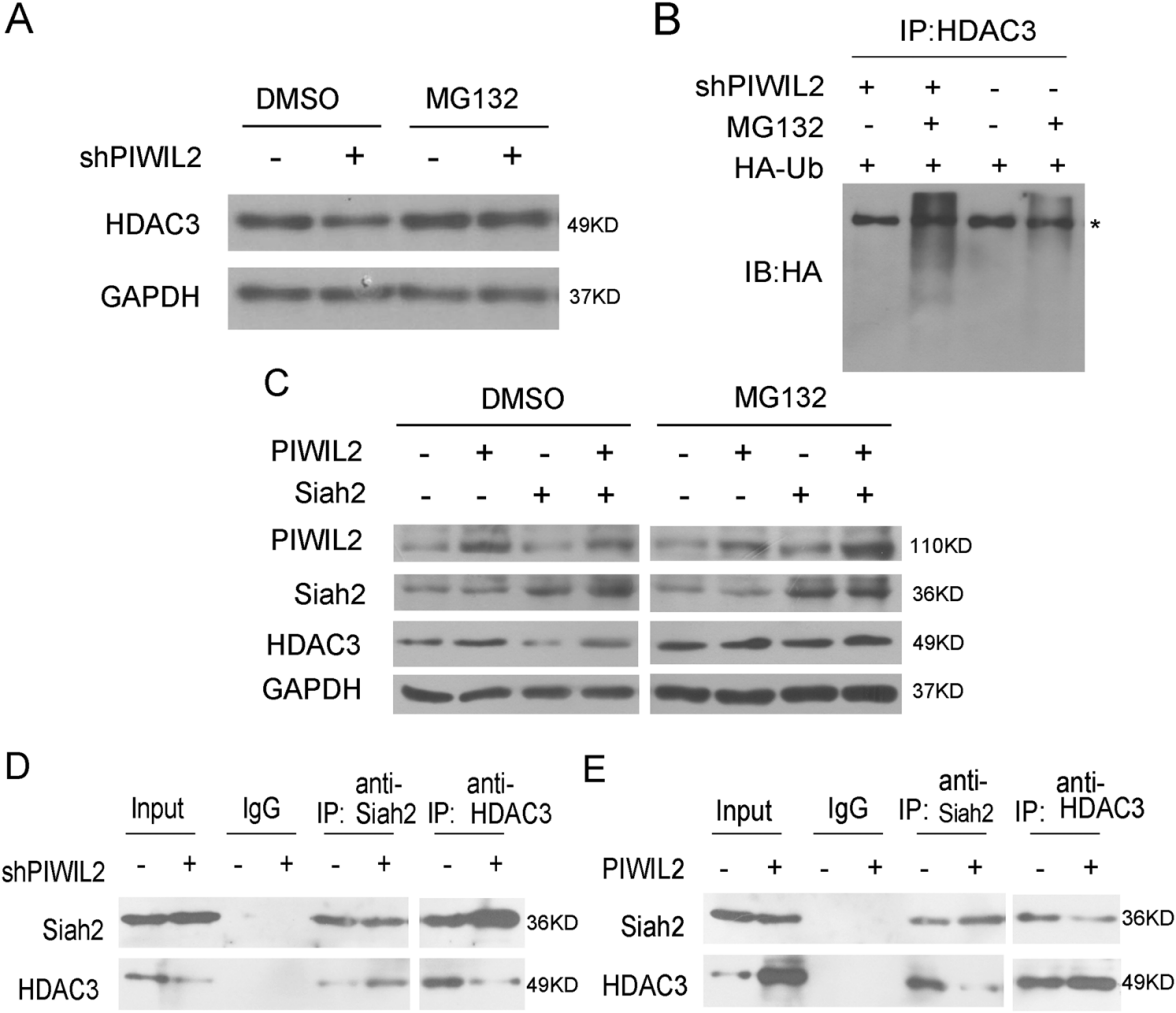
PIWIL2 inhibits the ubiquitination and degradation of HDAC3 by preventing Siah2 from binding to HDAC3. **a** MG132-treatment recovers HDAC3 protein level reduced by shPIWIL2. Cells were transfected with shPIWIL2 for 48 h and then treated with MG132 (10 μM) or DMSO (negative control) for 6 h. **b** PIWIL2 knockdown enhances the ubiquitin-mediated degradation of HDAC3. Hela cells were transfected with HA-ubiquitin vector, followed by treatment of MG132 for 6 h. Cell lysates were subjected to anti-HDAC3 antibody for WB. The “ *” indicates a non-specific bind. **c** PIWIL2 reduces Siah2-mediated degradation of HDAC3. Cells were transfected with PIWIL2 alone or in combination with Siah2, followed by a treatment with MG132 or DMSO as control for 6 h. **d**, **e** PIWIL2 reduces the interaction between Siah2 and HDAC3. Cells were transfected with PIWIL2 vector or PIWIL2 shRNA vector, and then analyzed by co-immuoprecipitation assays

The most common mechanism of targeting proteins for degradation by proteasome depends on poly-ubiquitination. So we studied whether PIWIL2 knockdown could enhance HDAC3 degradation via proteasome pathway. HDAC3 levels were determined in PIWIL2 knockdown cells in the presence or absence of the proteasome inhibitor MG132 (Fig. 3a). HDAC3 protein level reduced by shPIWIL2 can be recovered by MG132-treatment. Cells were transfected with HA-ubiquitin vector and treated with MG132 or DMSO, and then cell lysates were collected for Co-IP with anti-HDAC3 antibody, followed by WB assay with anti-HA antibody. As shown in Fig. 3b, knockdown of PIWIL2 enhanced the ubiquitination and degradation of HDAC3.

Since Siah2 has been implicated in the ubiquitination of HDAC3^30^, we doubt whether there is a competition between the PIWIL2 and Siah2 for binding to HDAC3. As shown in Fig. 3c, PIWIL2 reduced Siah2-mediated degradation of HDAC3 when cells were transfected with Siah2 alone or in combination with PIWIL2. MG132-treatment recovered the effect of both Siah2 and PIWIL2 on HDAC3 level.

We subsequently studied the effects of PIWIL2 overexpression or knockdown on the interaction between HDAC3 and Siah2. As shown in Fig. 3d, e, PIWIL2 reduced the binding of Siah2 with HDAC3 without significant effect on Siah2 level.

Collectively, these data suggest that PIWIL2 inhibits the Siah2-mediated ubiquitination and degradation of HDAC3.

### PIWIL2 facilitates the phosphorylation of HDAC3

**Fig. 4:**
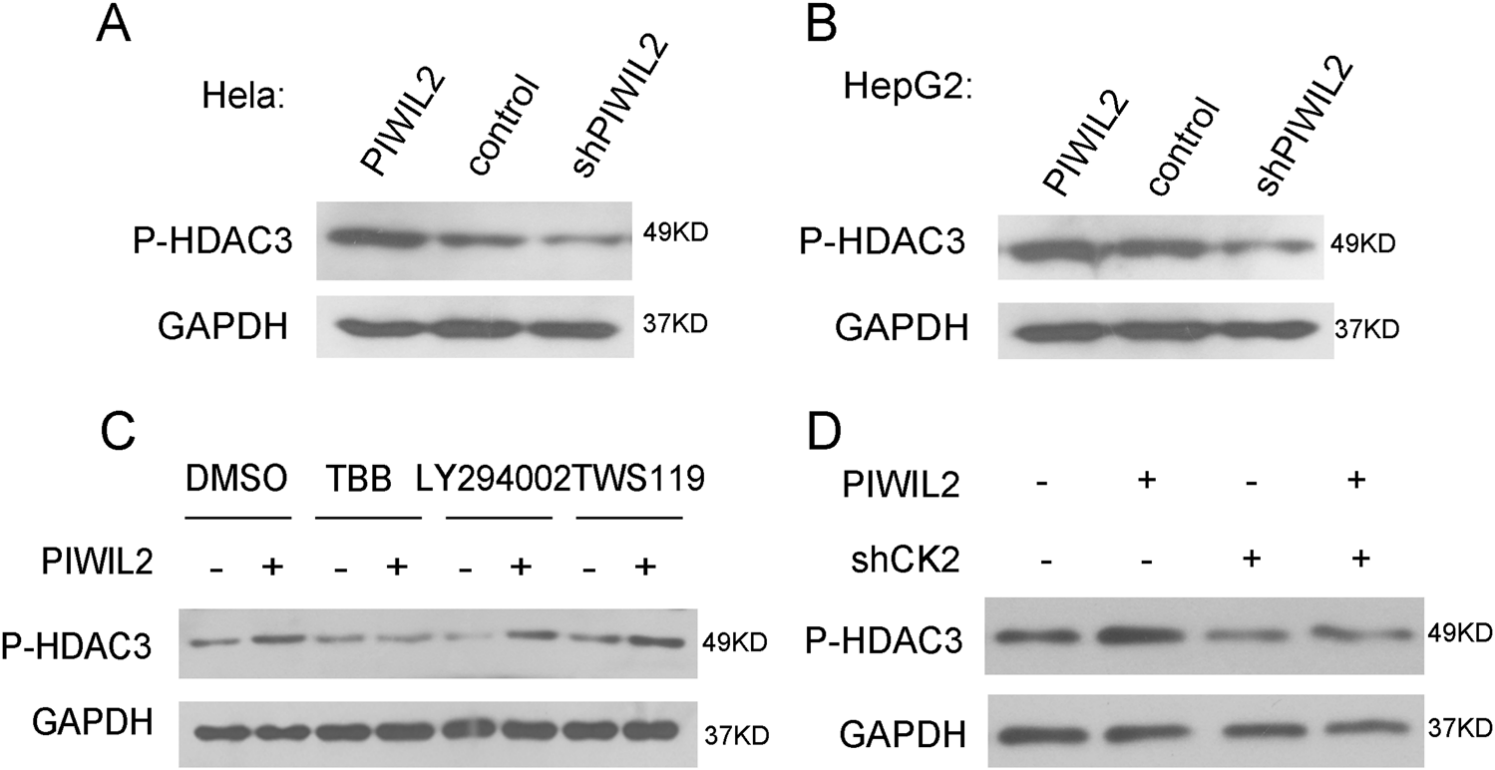
PIWIL2 promotes HDAC3 phosphorylation via CK2. **a**, **b** Hela and HepG2 cells were transfected with PIWIL2, shPIWIL2 or control vectors, respectively, followed by western blotting with antibody against P-HDAC3. **c** Treatment with CK2 inhibitor TBB abolishes the up-regulation of P-HDAC3 by PIWIL2. Hela cells transfected with PIWIL2 vector or control vector were treated with CK2 inhibitor TBB, PI3K inhibitor LY294002 or GSK3β inhibitor TWS119 for 6 h. **d** Knockdown of CK2 (shCK2α and shCK2α’) blocks PIWIL2 from upregulating P-HDAC3

The activity of HDAC3 can be influenced by its phosphorylation (S424) or its interaction with NcoR and SMRT^20,21^. Thus, we next examined whether PIWIL2 has an effect on the above mentioned HDAC3 activity. Knocking-down of PIWIL2 resulted in decreased HDAC3 phosphorylation (S424) levels, while overexpression of PIWIL2 had an opposite effect in both Hela and HepG2 cells (Fig. 4a, b). However, overexpression of PIWIL2 had no effect on the interaction between HDAC3 and NcoR or SMRT (data not shown).

PI3K-AKT, GSK3β, and CK2 were previously shown to phosphorylate HDAC3 in different types of cells^21,31,32^. We therefore examined whether PIWIL2 regulated HDAC3 phosphorylation through one of these pathways. Cells were treated with PI3K inhibitor LY294002, GSK-3βinhibitor TWS119 or CK2-specific inhibitor 4,5,6,7-tetrabromobenzotriazole (TBB), respectively, and HDAC3 phosphorylation levels were assessed. While LY294002 and TWS119 treatment had no effect on the role of PIWIL2 influencing phosphorylation of HDAC3, TBB treatment or shRNA against CK2 (shCK2α and shCK2α’) efficiently abolished the effect of PIWIL2 on HDAC3 phosphorylation in Hela or HepG2 cells (Fig. 4c, d).

### PIWIL2 promotes the combination of HDAC3 and CK2α

**Fig. 5:**
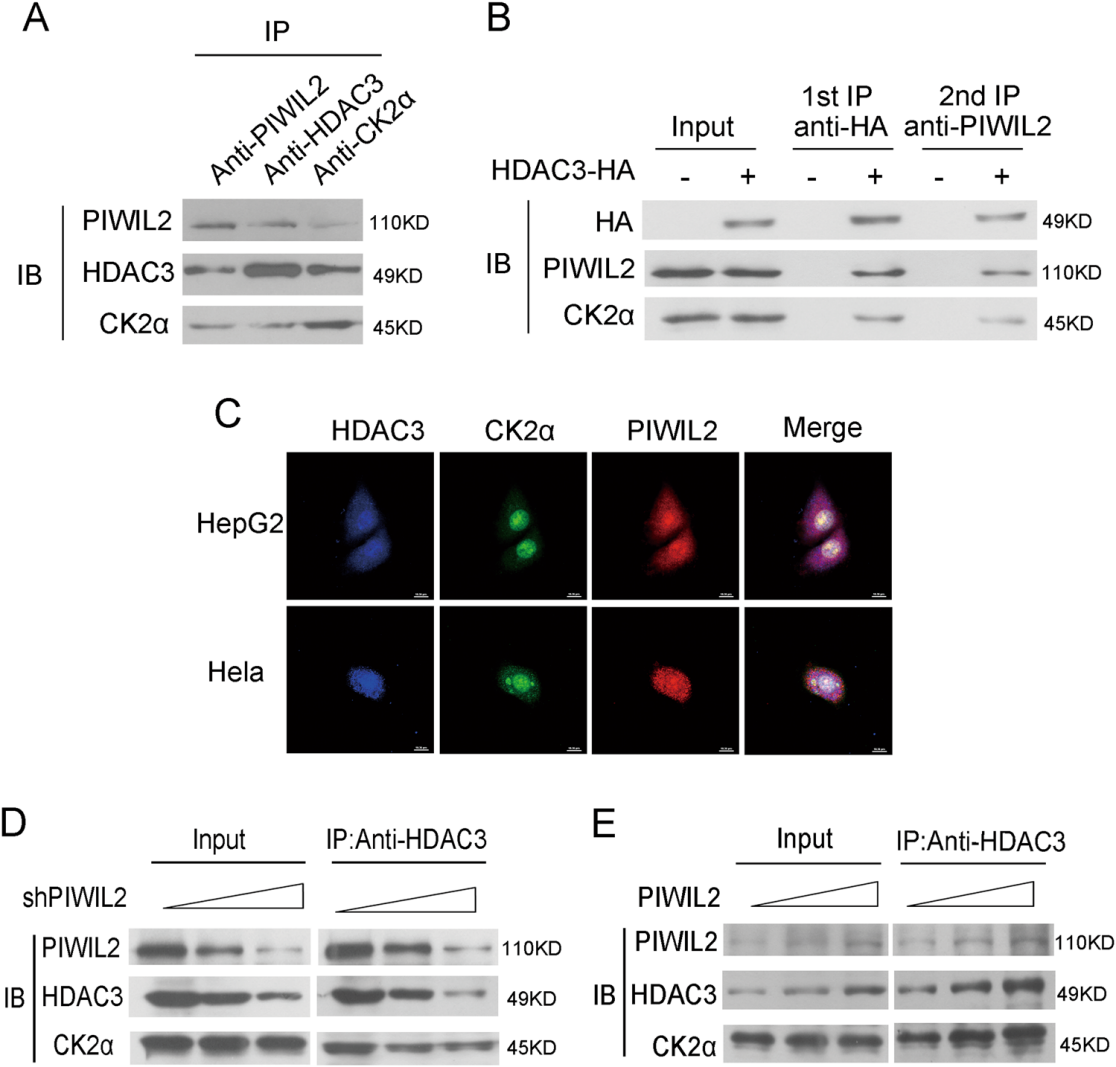
PIWIL2 enhances formation of CK2α-HDAC3 complex to phosphorylate HDAC3. **a** Co-immunoprecipitation assays showed the interaction of PIWIL2, CK2α, and HDAC3. **b** Cell lysates were immunoprecipitated with anti-HA antibody, and then a secondary immunoprecipitation assay was performed with anti-PIWIL2 antibody. **c** Immunofluorescence assays showed the co-localization of PIWIL2, CK2α, and HDAC3. **d**, **e** PIWIL2 facilitates CK2α binding with HDAC3 to phosphorylate HDAC3

As the result showed that only CK2 is involved in phosphorylation of HDAC3 induced by PIWIL2, we analyzed the relationship between PIWIL2 and CK2. Co-immunoprecipitation assays showed that PIWIL2 also had an interaction with CK2α, which may suggest that PIWIL2 formed a complex with CK2α and HDAC3 (Fig. 5a). This hypothesis was confirmed by a two-step IP assay (Fig. 5b). For further demonstration, immunofluorescence using antibodies against these three proteins was performed with a confocal microscope. The result showed that PIWIL2, HDAC3, and CK2α had co-localization in cell nucleus (Fig. 5c). Overexpression or knockdown of PIWIL2 had no significant effect on CK2α (Fig. 5d, e). Furthermore, co-immunoprecipitation assays suggested that PIWIL2 facilitated CK2α binding with HDAC3. These results indicate that PIWIL2 has a promotion on CK2 mediated HDAC3 phosphorylation by facilitating the association of CK2α and HDAC3.

### PIWIL2 stimulates cell proliferation and suppresses apoptosis via HDAC3

**Fig. 6:**
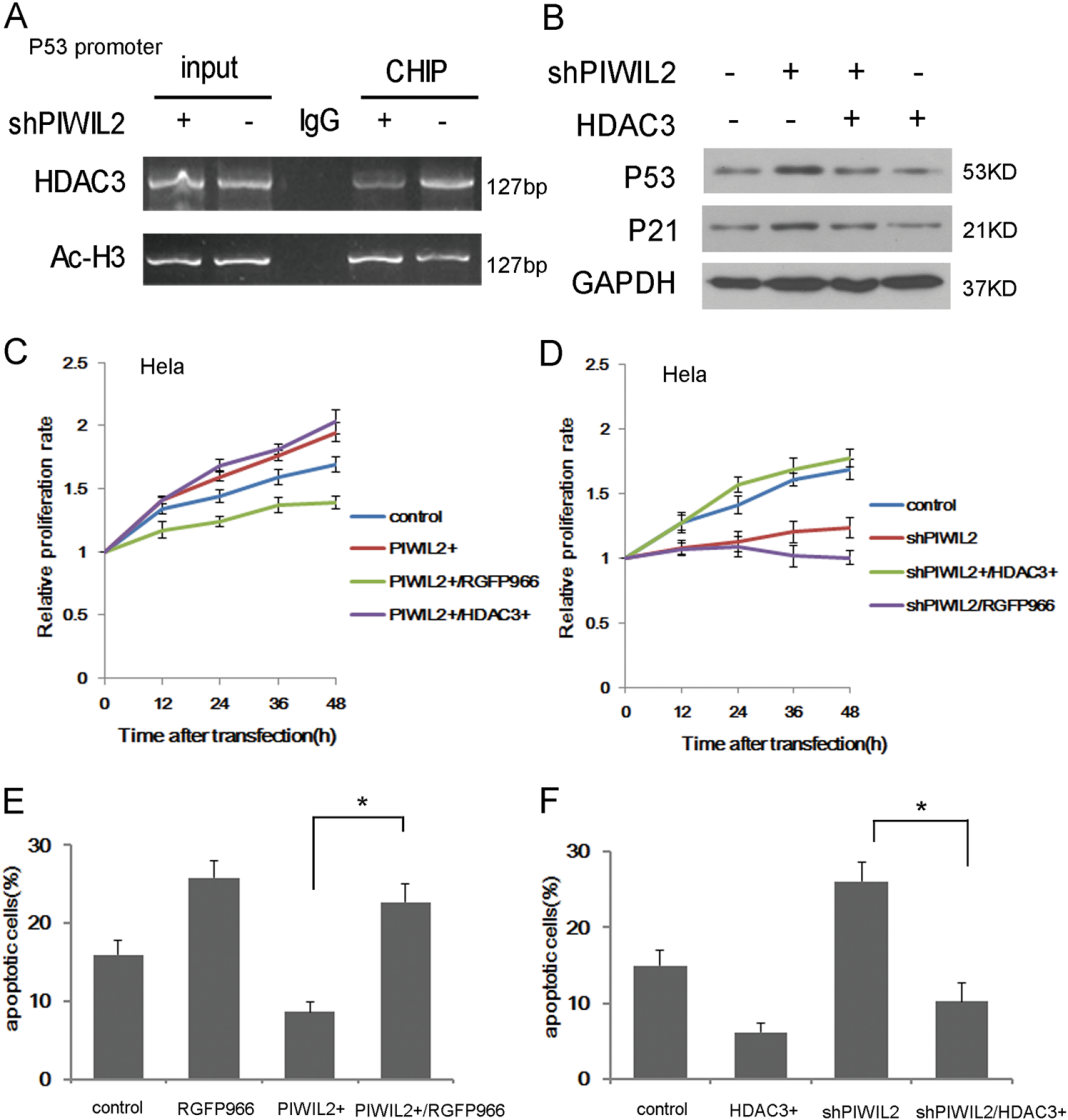
HDAC3 is involved in the role of PIWIL2 on cancer cell proliferation and apoptosis. **a** PIWIL2 knockdown reduces the binding of HDAC3 and increases Ac-H3 level on P53 promoter. **b** HDAC3 overexpression recovers the up-regulation of p53 and p21 by PIWIL2 knockdown. **c**, **d** In Hela cells, HDAC3 inhibition eliminates the effect of PIWIL2 on cell proliferation while HDAC3 overexpression recovers the impact of PIWIL2 knockdown on cell proliferation (*P* < 0.05). **e**, **f** HDAC3 inhibition eliminates the inhibition role of PIWIL2 on cell apoptosis. (*P* < 0.05)

Chromatin immunoprecipitation (CHIP) assay showed that PIWIL2 knockdown reduced the binding of HDAC3 and increased Ac-H3 level on P53 promoter (Fig. 6a). HDAC3 overexpression recovers the up-regulation of p53 and p21 induced by PIWIL2 knockdown (Fig. 6b). Next, we determined the effects of HDAC3 silencing on the role of PIWIL2 in cell proliferation and apoptosis. As shown in Fig. 6c, d, overexpression of PIWIL2 induced an increase in cell proliferation of Hela cells after 48 h transfection in comparison with control group. But inhibition of HDAC3 abolished the effect on promoting proliferation that caused by PIWIL2. We also observed that HDAC3 inhibition abolished the inhibition role of PIWIL2 on cell apoptosis, while overexpression of HDAC3 reduced the promotion of PIWIL2 knockdown on cell apoptosis (Fig. 6e, f).

## Discussion

HDACs are critical regulators in cell growth, apoptotic programs, and differentiation^33,34^. HDAC3, a member of HDACs family, overexpressed in various cancer cells, has been shown to regulate proliferation and apoptosis of cancer cells^14,18,35^. Previous study showed that overexpression of HDAC3 inhibited P53, P27, and Bax gene transcriptions via H3K9 deactylation and reduced basal and butyrate-induced p21^14,18^. However, factors that regulate HDAC3 in cancer remain largely unclear.

Emerging roles of PIWIL2 in cancer showed that PIWIL2 is involved in the proliferation, apoptosis, and migration of cancer cell. Our previous researches have shown that PIWIL2 promoted tumorigenesis and tumor development through many different ways. For example, PIWIL2 exerts a negative regulation on TGF-β signaling and suppresses p53 by inducing STAT3 phosphorylation^36,37^. We also found that PIWIL2 can bind to specific location of gene^27,38^, which prompted that PIWIL2 may have an epigenetic regulation on tumorigenesis.

Our results showed that PIWIL2 can interact with HDAC3 (Fig. 1) and stabilizes HDAC3 protein (Fig. 2) specifically in class I HDACs. PIWI domain of PIWIL2 was responsible for its interaction with HDAC3. C-terminal region of HDAC3 is necessary for the binding between HDAC3 and PIWIL2. All class I HDACs have a highly conserved HDAC domain, but their C terminus are very different. The finding suggested that PIWIL2 specifically regulates HDAC3 among class I HDACs.

As shown in Fig. 3, PIWIL2 stabilized HDAC3 by suppressing ubiquitin-mediated degradation. Siah2 has been reported as an E3 ubiquitin ligase that mediated the proteasome degradation of HDAC3^30^. We further showed that PIWIL2 binding to HDAC3 can interfere with the association of HDAC3 and Siah2 (Fig. 3d, e). Thus, association of PIWIL2 and HDAC3 prevented HDAC3 from interacting with Siah2 and subsequently its destruction.

In addition, HDAC3 can be phosphorylated by CK2, GSK3β or PI3K-AKT in different cell types and phosphorylation of HDAC3 can enhance its enzyme activity^21,31,32^. Our present study revealed that PIWIL2 knockdown reduced HDAC3 phosphorylation via CK2 (Fig. 4).

Furthermore, PIWIL2 can promote the interaction between HDAC3 and CK2α, leading to increase of HDAC3 phosphorylation by CK2α. Coimmunoprecipitation assay, immunofluorescence assay, and two-step IP assay further showed that PIWIL2, HDAC3, and CK2α could form a protein complex. PIWIL2 enhances the binding of CK2α with HDAC3. Thus, our results showed that PIWIL2 could modulate the activity of HDAC3 by promoting CK2α to combine with HDAC3 and phosphorylate HDAC3 (Fig. 5).

In tumor cells, enhanced HDACs expression or activity can result in transcription repression of genes that have a promotion on cell apoptosis and an inhibition on cell proliferation. As PIWIL2 promotes the stabilization and phosphorylation of HDAC3, less HDAC3 can be observed in binding with P53 promoter when PIWIL2 was knockdowned, leading to increased Ac-H3 level on P53 promoter (Fig. 6). Thus, the level of p53 and its downstream protein p21 were up-regulated by PIWIL2 inhibition. Knockdown of HDAC3 reduced the effect of PIWIL2 on cancer cell proliferation or apoptosis, indicating that HDAC3 was involved in the role of PIWIL2 on cancer proliferation and apoptosis.

**Fig. 7:**
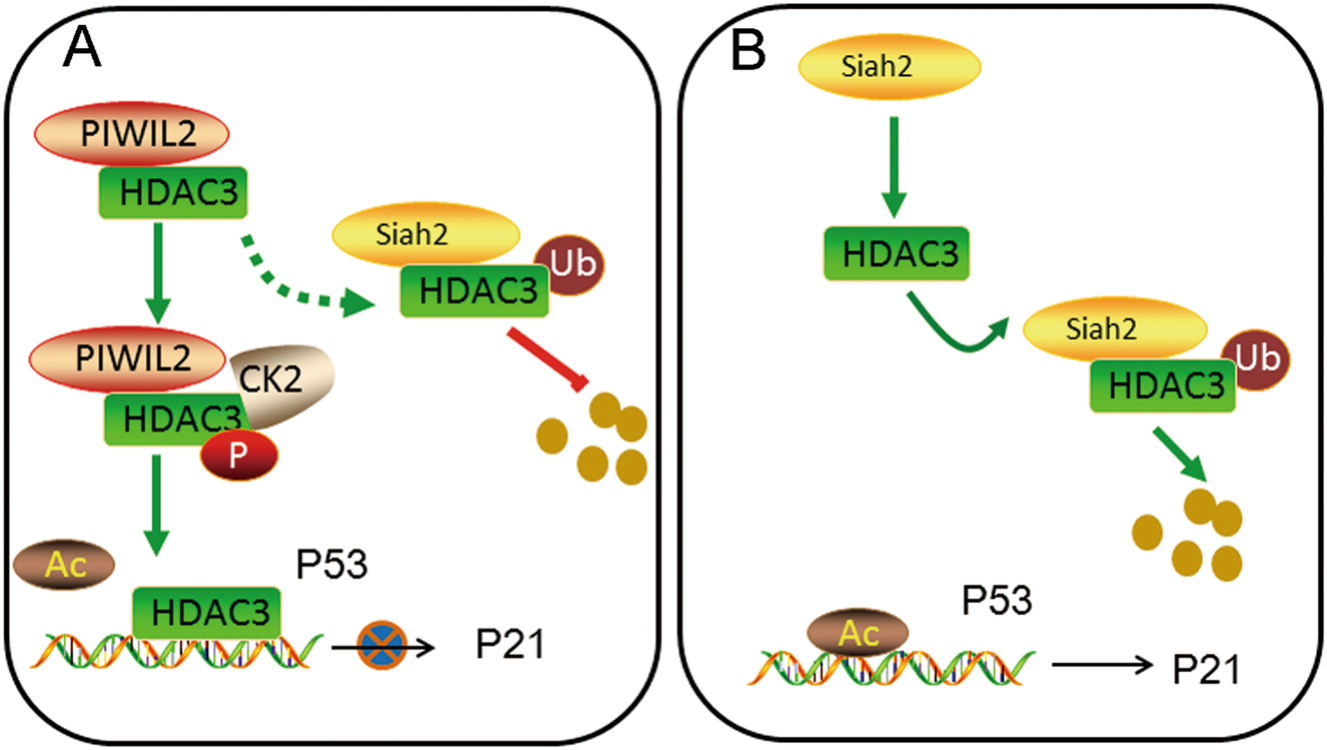
Model of PIWIL2 on proliferation of cancer cell via HDAC3. **a** In WT cancer cells (at least Hela and HepG2), PIWIL2 stabilizes HDAC3 by inhibiting Siah2-mediated degradation. PIWIL2 binding enhances CK2-mediated phosphorylation of HDAC3. As a result, the expression of p53 is reduced followed by a decrease of p21. **b** In PIWIL2 knockdown cells, p53 has a relative high expression level and p21 as well

Taken together, the present study shows that PIWIL2 regulates HDAC3 through two different ways: first, PIWIL2 interacts with HDAC3 and stabilizes HDAC3 from ubiquitin-mediated degradation by competitive association with E3 ubiquitin ligase Siah2; second, PIWIL2 promotes HDAC3 phosphorylation via CK2α. Through cutting down the stabilization and phosphorylation of HDAC3, PIWIL2 inhibition decreased the binding of HDAC3 on P53 promoter and enhanced Ac-H3 on P53 promoter followed by increasing p53 and p21 level. The reduced stabilization and phosphorylation of HDAC3 caused by PIWIL2 knockdown inhibits cancer cell proliferation and increases cancer cell apoptosis (Fig. 7).

In summary, our results reveal that PIWIL2 interacts with HDAC3 and promotes the stability and phosphorylation of HDAC3. We reveal a novel role that PIWIL2 plays in epigenetic regulation in tumorigenesis.

## Materials and methods

### Plasmids and antibodies

CDS encoding HDAC3, CK2α, Siah2, and HDAC3 deletion mutants were synthesized and inserted into pcDNA3.1 vector. A set of deletion mutants and wide type PIWIL2 were constructed in our previous study^37^. shRNA against PIWIL2, HDAC3, CK2 were synthesized by GenePharma company (Shanghai, China). shRNA for PIWIL2, HDAC3, and CK2 were synthesized and cloned into pGPU6/GFP/Neo, and the target sequences of these shRNA were as follows:

PIWIL2 shRNA (shPIWIL2): 5′-CTATGAGATTCCTCAACTACAGAAG-3′

HDAC3 shRNA(shHDAC3): 5′-GCTGGTAGAAGAGGCCATTAG-3′

CK2α shRNA(shCK2α): 5′-ATTACCTGCAGGTGGAATATT-3′

CK2α’ shRNA(shCK2α’): 5′-CCTCACAATGTCATGATAGAT-3′

Antibodies against each named protein were: PIWIL2 (Santa Cruz, CA, USA); HDAC1, HDAC2, and HDAC3 (Cell signaling technology, MA, USA); HDAC8, P-HDAC3 (EnoGene, Nanjing, China); Siah2 (Proteintech, IL, USA); CK2α (Abgent, Suzhou, China); p53, HA, Myc (Santa Cruz, CA, USA); p21, GAPDH (Cell signaling technology, MA, USA).

### Cell culture and transfection

Human cervical cancer cell line Hela and hepatocellular carcinoma cell line HepG2 were maintained in our laboratory. Cells were cultured at 37 °C, 5%CO_2_ in DMEM with 10% fetal bovine serum. The transfection was performed with jetPRIME^TM^ (Polyplus-transfection, SA, France) according to the manufacturer’s instruction, and transfected cells were harvested at 48 h post transfection. Proteasome inhibitor MG132 and cycloheximide (CHX) were purchased from Sigma (St. Louis, MO, USA). For CHX treatment, cells were treated with CHX (50 μM) for indicated time. For MG132 treatment, cells were treated with MG132 (10 μM) for 6 h before harvesting. For treatment of Kinase inhibitor, cells were pretreated with TBB (50 μM), LY294002 (50 μM) or TWS119 (10 μM) for 6 h. HDAC3 inhibitor RGFP966 (5 μM) were purchased from MCE (Med Chemexpress, LLC, USA).

### Immunofluorescence

Cells cultured on 24-well chamber slides were fixed in 4% paraformaldehyde for 15 min, treated with 0.5% TritonX-100 in PBS for 10 min and incubated with 1% BSA for 30 min at RT. For detection of specific protein, cells were incubated with primary antibodies overnight at 4 °C and followed with secondary antibodies for 1.5 h at RT. All secondary antibodies (Alexa Fluor® 488, Alexa Fluor®555, Alexa Fluor®350) were purchased from Thermo Fisher Scientific Inc. (Thermo Fisher Scientific, CA, USA). The nucleus was detected with DAPI (Sigma-Aldrich, St. Louis, MO, USA) staining. Images were acquired with laser scanning confocal microscope (Olympus, Tokyo, Japan).

### Immunoprecipitation

Cells were collected and lysed in IP lysis buffer (150 mM NaCl, 50 mM Tris pH8, 1% Triton X-100, 1% NP-40) supplemented with protease and phosphatase inhibitors, incubated on ice for 20 min, and cleared by centrifugation at 12,000 rpm at 4 °C for 10 min. Total protein lysate (1 mg) was immunoprecipitated with antibody (anti-PIWIL2, HDAC3 or IgG as control antibodies) and incubated on a rotator overnight at 4 °C and followed by incubation with protein A + G agarose beads (Beyotime Biotechnology, Shanghai, China) for 2 h. Immune complexes were eluted from the agarose beads and analyzed by SDS-PAGE followed by immunoblot analysis.

### Immunohistochemistry

Tissue microarray slides of cervical cancer (Shanghai Outdo Biotech, Shanghai, China) were deparaffinized and rehydrated with xylene graded alcohol solutions. Sections were incubated with primary antibodies overnight at 4 °C, and subsequently with corresponding secondary antibody (Thermo Fisher Scientific, CA, USA). After staining with DAB, slides were counterstained with hematoxylin. The staining intensity was analyzed as follows: negative = 0, weak = 1, moderate = 2, strong = 3. The percentage of positive tumor cells was graded as follows: none (0), <25% (1), 26–50% (2), 51–75% (3), 76–100% (4). Therefore, score of each tissue was 0–12 by multiplying the score of staining intensity with score of percentage of staining.

### Western blotting

Cells were lysed with universal protein extraction lysis buffer (Bioteke, Peking, China) which contained protease inhibitors (Roche, Basel, Switzerland). Protein lysates were run on 8–12% SDS PAGE gels and then transferred onto a PVDF membrane (Millipore, Billerica, MA, USA). The membrane was incubated at room temperature for 1 h in 3% BSA and then blotted with specific primary antibody overnight at 4 °C. After incubation for 1 h at room temperature in the HRP-labeled secondary antibody, the membrane was detected with chemiluminescence Western blot detection system (Millipore, Billerica, MA, USA).

### CHIP assay

CHIP assays were performed using a CHIP assay kit (Beyotime Biotechnology, Shanghai, China) according to the manufacturer’s instruction. Cells were fixed in 1% formaldehyde for 10 min, and then crosslinking was stopped by adding 125 mM glycine. Cell lysates were sheared by sonication in 1% SDS lysis buffer to generate chromatin fragments, and immunoprecipitated with 2 µg antibodies overnight at 4 ˚C with antibodies specific to HDAC3, Ac-H3 or IgG as a negative control. Antibody-protein-DNA complex were precipitated with protein A-agarose beads at 4 ˚C for 2 h. Input or the associated DNA fragment were purified and analyzed by PCR with specific primer for P53 promoter [(5′-ATT CTG CCC TCA CAG CTC TGG CT-3′ (sense) and 5′-CCG GAG GAA GCA AAG GAA ATG G-3′ (antisense)]^37^. Same amount (10 µl) of PCR production in each group was used for agarose gel electrophoresis.

### RNA extraction and real-time PCR

Total RNA was extracted from cells using a RNA Rapid Extraction Kit (BioTeke Corporation, Peking, China) and was reversely transcribed using a reverse transcription kit (Thermo Fisher Scientific, MA, USA) according to the manufacturer’s instructions. The subsequent Real-time PCR was performed using the SYBR® Green Master Mix (Bio-Rad Laboratories, CA, USA) with GAPDH as an internal control for normalization. Differential expression was calculated according to the 2^-△△CT method and statistically evaluated.

### Proliferation and apoptosis assay

The cell proliferation was determined with WST-8 Cell Counting Kit-8 (Beyotime Biotechnology, Shanghai, China). Briefly, cells were seeded at 1 × 10^4^ cells in a 96-well plate. After incubation for 24 h at 37 °C, cells were transfected with specific plasmid. After 6,12, and 24 h, cells in each well of the plates were then treated with 10 µl of assay reagent in the dark for 90 min at 37 °C. The absorbance was recorded at 450 nm with a micro plate reader (BioTek, Vermont, USA). The apoptosis of cells were detected by an Annexin V/PI Apoptosis Detection Kit (US Everbright, California, USA) according to the manufacturer’s instruction. Apoptotic rates were analyzed by a Coulter Epics XL flow cytometer (Beckman, Urbana, IL, USA).

### Statistical analysis

Each experiment was repeated three times. Statistical analyses were performed using SPSS version 17.0 (IBM Company, Chicago, IL, USA). The differences between two groups were analyzed using a Student’s *t*-test. A *P*-value of <0.05 was considered statistically significant.
